# Causal Gene Identification and Biomarker Prioritization in Periodontitis via Integrative Multiomics and Mendelian Randomization

**DOI:** 10.1155/mi/6044837

**Published:** 2025-11-24

**Authors:** Haokun Mo, Lulu Chen, Shanshan Ren, Yue Wu, Ai Tian

**Affiliations:** ^1^School of Stomatology, Guizhou Medical University, Guiyang, Guizhou, China; ^2^Medical Collaboration Department, Affiliated Hengyang Hospital of Hunan Normal University and Hengyang Central Hospital, Hengyang, Hunan, China; ^3^Department of Prosthodontics, The Affiliated Stomatological Hospital of Guizhou Medical University, Guiyang, Guizhou, China

**Keywords:** biomarkers, Mendelian randomization, periodontitis, SMR, WGCNA

## Abstract

**Introduction:**

Periodontitis is a common inflammatory disease that compromises oral and systemic health. This study aimed to elucidate its molecular mechanisms and identify potential biomarkers for early diagnosis and precision treatment.

**Methods:**

We integrated genome-wide association study (GWAS) and transcriptomic data from periodontitis patients and healthy controls. Summary data-based Mendelian randomization (SMR) and the heterogeneity in dependent instruments (HEIDI) test were used to identify genetically associated genes. Differentially expressed genes (DEGs) were identified using LIMMA, and weighted gene co-expression network analysis (WGCNA) revealed disease-related gene modules. Candidate biomarkers were prioritized through intersection analysis and evaluated using five machine learning algorithms. Causal relationships were further validated by two-sample Mendelian randomization (TSMR). Functional enrichment was assessed via gene set enrichment analysis (GSEA) and gene set variation analysis (GSVA), and immune infiltration was analyzed using CIBERSORT.

**Results:**

SMR identified 360 gene-trait associations, with 320 passing the HEIDI test, corresponding to 294 unique genes. DEGs were enriched in immune and neuronal development pathways. WGCNA uncovered nine gene modules associated with periodontitis. Intersection and machine learning analyses identified five key biomarkers—GPX2, IGKV2D-30, CD34, GSTA4, and NYNRIN—with strong predictive performance, validated by MR analysis (*p* < 0.05). Immune infiltration analysis revealed increased regulatory T cells, activated mast cells, and neutrophils, and decreased memory B cells and resting mast cells in periodontitis, with biomarker expression levels showing significant immune correlations.

**Conclusion:**

This integrative multiomics analysis uncovers causal genes and robust biomarkers involved in periodontitis pathogenesis, providing new insights for early detection and individualized treatment strategies. Further experimental validation is needed to confirm their functional roles in disease progression and therapeutic potential.

## 1. Introduction

Periodontitis is a chronic multifactorial inflammatory disease that begins with the formation of a dental plaque biofilm [1–4]. However, it is now widely accepted that the progression of periodontitis is primarily driven by an inappropriate host immune response and the sustained release of inflammatory mediators. Cytokines, such as IL-1β, IL-6, and TNF-α play a central role in the recruitment of immune cells, activation of osteoclasts, and destruction of periodontal tissues.

Moreover, chronic inflammation in periodontitis may have systemic consequences and has been implicated in the development of several noncommunicable diseases. For example, recent studies have shown correlations between periodontitis and cancer [5], including lung [6, 7], esophageal [8–10], and gastric adenocarcinoma [10, 11]. It is thought that systemic inflammatory responses triggered by periodontal disease may promote tumor progression. Similarly, periodontitis is associated with cardiovascular diseases [12, 13], liver diseases [14], and diabetes [15, 16], likely mediated by shared inflammatory mechanisms and immune dysregulation. Evidence also suggests that periodontal treatment can improve both oral and systemic health outcomes, such as reducing postoperative pneumonia and possibly slowing the progression of other chronic diseases [17].

Given this complex interplay between local and systemic inflammation, understanding the immune-inflammatory mechanisms and identifying molecular targets are critical for developing new diagnostic and therapeutic strategies. However, traditional research methods often struggle to reveal the causal relationships between genetic variation and disease phenotypes.

With recent advances in bioinformatics and genomics, methods, such as genome-wide association study (GWAS)-based summary data-based Mendelian randomization (SMR) and two-sample Mendelian randomization (TSMR) have become powerful tools to uncover genes causally involved in disease development [18, 19]. These approaches help infer whether gene expression changes contribute to disease onset or progression. Furthermore, differential expression analysis and weighted gene co-expression network analysis (WGCNA) allow for the identification of key regulatory genes and network modules associated with periodontitis [20]. Machine learning algorithms are also increasingly used to detect complex patterns in high-dimensional data and to build predictive diagnostic models [21].

This study aims to apply an integrative approach combining Mendelian randomization (MR), transcriptome analysis, WGCNA, and machine learning to systematically explore the pathogenesis of periodontitis, identify novel biomarkers, and establish a reliable diagnostic model for early detection and treatment.

## 2. Materials and Methods

### 2.1. SMR

We performed SMR analysis using the SMR software (v1.03), with parameters set according to the developers' recommendations [18, 22]. SNPs were selected as instrumental variables (IVs) based on their association with gene expression levels at a genome-wide significance threshold (*p* < 5 × 10^−8^), and clumped using linkage disequilibrium (LD) criteria (*r*^2^ < 0.05 within 1 Mb window) to ensure independence. To distinguish true pleiotropic effects from linkage, we applied the heterogeneity in dependent instruments (HEIDI) test, which evaluates whether the observed association between gene expression and the trait is due to a single causal variant. A HEIDI *p*-value ≥ 0.01 was considered to support a causal association, while results with significant heterogeneity (*p* < 0.01) were excluded to avoid LD confounding. To correct for potential multicollinearity bias arising from correlated SNPs, we used genotype data from the 1000 genomes project (European panel) as the LD reference and ensured that SNPs in high LD were appropriately filtered. Both summary-level GWAS data for periodontitis and complete eQTL datasets in PLINK binary format were provided as input. These strategies collectively improve the robustness of IV selection, pleiotropy filtering, and control of LD structure in the SMR framework.

### 2.2. Analysis Using the eQTL-Gen Database and Periodontitis GWAS Data

We also utilized eQTL (*p* < 1*e*–5) results from periodontitis GWAS and the eQTL-Gen database to run the SMR software. The eQTL-Gen database recently conducted a study on blood samples from 31,684 individuals [23]. This study employed periodontitis data from European samples, sourced from the FinnGen project (https://www.finngen.fi/en) [24]. This project, led by the University of Helsinki, obtained approvals from local institutional review boards and ethics committees for each study. The Centers for Disease Control and Prevention (CDC)/American Academy of Periodontology (AAP) used probe depth assessments or self-reports to classify periodontitis cases, which included 4784 periodontitis cases and 272,252 control cases. We utilized these data for SMR analysis.

### 2.3. Bulk Sequencing Data

We used the GSE223924 dataset as an independent training dataset, which comprises gingival tissue samples from 10 periodontitis patients and 10 healthy individuals. Additionally, the microarray datasets GSE10334 [25] and GSE16134 [26] were utilized as independent external validation datasets. These datasets contain gingival tissue data from 63 and 241 chronic periodontitis patients, and 64 and 69 healthy individuals, respectively.

### 2.4. Identification of Differential Genes

We normalized the raw expression matrix using R software and identified differentially expressed genes (DEGs) from the GSE223924 dataset using the limma R package [27], with adjusted *p*-values less than 0.05 and |log FC| greater than or equal to 1. We also created a clustering heatmap of the DEGs using R software.

### 2.5. WGCNA

WGCNA is a bioinformatics analysis method used to study gene association patterns, allowing for the clustering of genes with similar expression patterns and the exploration of relationships between modules and specific traits or phenotypes. We used the WGCNA R package [28] to construct a co-expression network. In this process, we included genes with adjusted *p*-values less than 0.05. First, we performed hierarchical clustering using the “hclust” function to exclude potential outliers. Next, we used the “pickSoftThreshold” function to select an appropriate soft-threshold power (β) to ensure that the gene expression relationships conformed to a scale-free network. We then used the “adjacency” function to convert the gene expression similarity matrix into an adjacency matrix based on the selected β value. Subsequently, we transformed the resulting adjacency matrix into a topological overlap matrix (TOM) to reduce the impact of noise and spurious associations. Finally, we detected modules using hierarchical clustering and dynamic tree cutting functions, and explored the relationships between modules and clinical traits of patients through Pearson correlation analysis (*p*-value less than 0.05).

### 2.6. Identification of Biomarkers Based on Five Machine Learning Methods

We used a Venn diagram to intersect the results of differential expression analysis, WGCNA, and SMR to identify genes for further machine learning analysis. We also performed machine learning analysis using marker genes obtained from the preliminary screening. First, we randomly divided the dataset into a training set and a validation set in a 7 : 3 ratio. Then, we constructed diagnostic models based on gene expression data using SVM, random forest (RF), XGBoost (XGB), generalized linear model (GLM), and K-nearest neighbors (KNN) algorithms. We primarily used the “caret” [29] and “DALEX” [30] R packages to optimize the model construction process and interpret the relationships between input and output variables. SVM classifies data by constructing a hyperplane and uses a regularization term to prevent overfitting [31]. RF is an ensemble learning method that performs classification or regression by constructing multiple decision trees and combining their results, effectively handling nonlinear effects and interactions between variables [32]. XGB is a gradient boosting tree algorithm that combines linear models and boosted tree models, efficiently handling large-scale datasets, and optimizing and regularizing the model during training to improve predictive performance [33]. GLM is a statistical model used to describe the relationship between dependent and independent variables by selecting appropriate distribution families and link functions, commonly used for tasks like linear regression and logistic regression [34]. KNN is a simple and intuitive classification algorithm that classifies new data based on the distance to the KNN in the training set, assigning the new data to the most frequently occurring category among the neighbors [35].

We then calculated the area under the receiver operating characteristic (ROC) curve (AUC) and accuracy to evaluate the classification ability of each model [36]. Finally, we selected the algorithm with the highest AUC and the smallest residuals to construct the final model. Residuals were defined as the difference between the observed values and the model's estimated values, with smaller residuals indicating a better fit of the model to the actual data. The top five most important genes identified by this algorithm were defined as potential biomarkers for periodontitis. We then searched for the chromosomal start and end positions of these biomarkers in the Ensemble genome database.

### 2.7. TSMR

TSMR analyses were conducted using summary-level data from the MRC IEU OpenGWAS project (https://gwas.mrcieu.ac.uk/) [37], including the following eQTL datasets: eqtl-a-ENSG00000176153 (GPX2), eqtl-a-ENSG00000174059 (CD34), eqtl-a-ENSG00000170899 (GSTA4), eqtl-a-ENSG00000205978 (NYNRIN), and eqtl-a-ENSG00000239571 (IGKV2D-30). SNPs were selected as IVs based on genome-wide significance (*p* < 5 × 10^8^), LD clumping (*r*^2^ < 0.001 within 10,000 kb), and presence in both exposure and outcome datasets. To mitigate potential biases due to multicollinearity, we applied stringent LD pruning and excluded SNPs with known pleiotropic associations based on external databases. Causal effects were estimated using the inverse-variance weighted (IVW) method as the primary approach, complemented by MR–Egger, weighted median, weighted mode, and simple mode methods [38–42]. Horizontal pleiotropy was assessed using the MR–Egger intercept (*p* > 0.05 indicating no pleiotropy), and heterogeneity was evaluated via Cochran's *Q* statistic. To further account for potential outlier effects and residual pleiotropy, we performed MR-PRESSO analysis. Our analysis incorporated *Q* statistics and MR–Egger as analogous sensitivity tools. All analyses were implemented in R using the TwoSampleMR and MRPRESSO packages.

### 2.8. Identification and Correlation of Immune Cell Infiltration in Disease

We employed the CIBERSORT algorithm, which utilizes linear support vector regression to deconvolute gene expression profiles and estimate the abundance of immune cells in RNA sequencing data [43]. Using the CIBERSORT algorithm in R, we calculated the proportions of 22 immune cell types across different immune patterns in the dataset. The immune cell composition of patient immune patterns was visualized using violin plots. The Wilcoxon rank-sum test was used to evaluate differences in immune cell proportions. Additionally, we analyzed the correlation between model genes and tumor-infiltrating immune cells, with a *p*-value less than 0.05 considered statistically significant.

### 2.9. Gene Set Enrichment Analysis (GSEA) and Gene Set Variation Analysis (GSVA)

To identify signaling pathways in the high and low expression groups of model genes in periodontitis, we selected an ordered gene list using the limma R package and performed GSEA on the GSE223924 dataset using the clusterProfiler R package [44]. Simultaneously, we conducted GSVA on GSE223924, with adjusted *p*-values less than 0.05, to explore pathways associated with the five model genes [45]. We downloaded the C2 and C5 gene sets “c2.cp.kegg.symbols.gmt” and “c5.go.symbols.gmt” from the Molecular Signatures Database (https://software.broadinstitute.org/gsea/downloads.jsp) and used the GSVA algorithm to comprehensively score each gene set, assessing potential biological functional changes in the two periodontitis groups.

### 2.10. Construction of Column Charts and Diagnostic Performance Evaluation

We constructed a diagnostic model using logistic regression analysis and visualized it by plotting a nomogram. The ROC curve was plotted, and the AUC was calculated using the “pROC” R package. Diagnostic performance was evaluated using AUC, calibration curves, and decision curve analysis (DCA). Finally, we validated the differential expression and predictive reliability of the biomarkers using the validation set.

### 2.11. Statistical Analysis

All statistical analyses were performed using R software (version 4.3.0). DEGs were identified using the limma package with Benjamini–Hochberg correction (adjusted *p*  < 0.05). WGCNA was conducted based on Pearson correlation to identify trait-related modules. MR analyses (SMR and TSMR) applied genome-wide significant SNPs (*p* < 5 × 10^−8^), with LD pruning and HEIDI tests to control for linkage and pleiotropy. In TSMR, IVW was the primary method, complemented by MR–Egger, weighted median, and MR-PRESSO for sensitivity analyses. For machine learning, the dataset was randomly split into training and validation sets (7 : 3), and model performance was evaluated using AUC and residuals. Immune infiltration differences were assessed using the Wilcoxon rank-sum test. GSEA and GSVA were performed using clusterProfiler and GSVA packages, respectively, with FDR correction. *p*-values < 0.05 were considered statistically significant unless otherwise specified. The study flow chart is presented in Supporting Information 1: Figure S1.

## 3. Results

### 3.1. SMR Results

**Figure 1:**
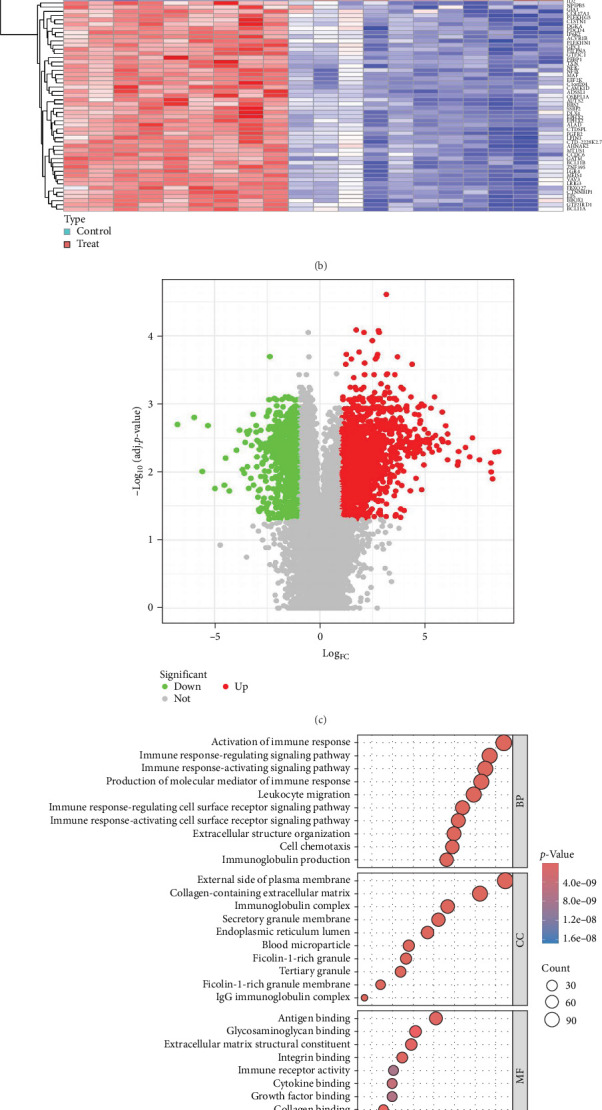
Functional enrichment analysis in periodontitis. (a) GO enrichment analysis results for periodontitis-related genes identified by SMR. (b) Heatmap illustrating the differentially expressed genes in the training set, with samples grouped by periodontitis and normal. (c) Volcano plot displaying the DEGs. (d) GO enrichment analysis results for DEGs significantly up-regulated in periodontitis. (e) GO enrichment analysis results for DEGs significantly downregulated in periodontitis.

A comprehensive analysis of the transcriptome in blood tissue revealed 360 gene-feature associations identified through univariate SMR analysis, with FDR *q*-value <0.05. No associations were excluded after multi-SNPs SMR analysis. Among these 360 associations, 20 did not pass the HEIDI test, resulting in 340 significant associations, which mapped to 294 genes (Supporting Information 2: Table S1). The top three genes positively correlated with periodontitis were identified as death effector domain containing 2, interferon induced protein 44 like, and bestrophin 3, while the top three negatively correlated genes were TTK protein kinase, solute carrier family 16 member 10, and DAB2 interacting protein. Gene Ontology (GO) enrichment analysis of biological processes revealed that these genes were primarily enriched in lymph vessel morphogenesis, regulation of metaphase plate congression, vesicle transport along microtubule, regulation of interleukin-2 production, and interleukin-2 production (Figure 1a).

### 3.2. Identification and Functional Enrichment Analysis of DEGs

Using the LIMMA package, we conducted an analysis of DEGs between periodontitis and healthy controls in the GSE223924 dataset (|Log_FC_| > 1, *p*  < 0.05). A total of 2097 DEGs were identified, with 718 downregulated and 1379 upregulated genes (Supporting Information 3: Table S2). Heatmaps and volcano plots illustrated these upregulated and downregulated genes (Figure 1b,c). GO functional enrichment analysis revealed that upregulated DEGs were significantly enriched in leukocyte migration, production of molecular mediators of immune response, immune response-activating signaling pathway, immune response-regulating signaling pathway, and activation of immune response (Supporting Information 4: Table S3 and Figure 1d). Conversely, downregulated DEGs were predominantly enriched in axonogenesis, regulation of neuron projection development, skin development, axon development, and epidermis development (Supporting Information 5: Table S4 and Figure 1e).

### 3.3. Periodontitis-Related Genes Unearthed by WGCNA

**Figure 2:**
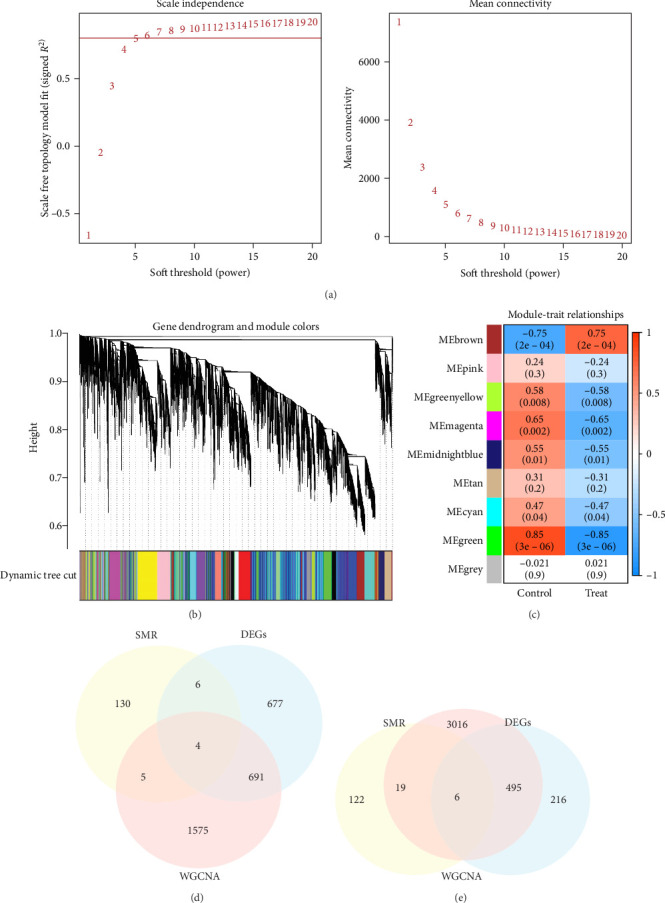
Identification of hub genes and markers for periodontitis using WGCNA. (a) Analysis of scale independence and mean connectivity for different soft-thresholding powers (β) ranging from 1 to 20. (b) Cluster dendrogram based on weighted correlation coefficients, with genes showing similar expression patterns grouped into co-expression modules, each module represented by a unique color. (c) Heatmap illustrating the correlation between module eigengenes (MEs) and clinical traits, including stemness subtypes. (d) Overlap of genes positively associated with periodontitis identified through SMR, upregulated genes in periodontitis from differential expression analysis, and genes positively associated with periodontitis from WGCNA. (e) Overlap of genes negatively associated with periodontitis identified through SMR, downregulated genes in periodontitis from differential expression analysis, and genes negatively associated with periodontitis from WGCNA.

WGCNA analysis of the GSE223924 dataset yielded gene modules associated with periodontitis. Samples with no outliers were excluded from the WGCNA analysis. The selection of soft-thresholding power showed that the gene correlation was most consistent with a scale-free distribution when *β* = 5. Subsequently, nine modules were identified by merging modules with a feature factor greater than 0.25 and setting the minimum gene number per module to 200 (Figure 2a,b). We found that the MEbrown module (*r* = 0.75, *p*=2e − 04) was positively correlated with periodontitis. Genes within the MEbrown module were further analyzed, revealing 2279 genes positively correlated with periodontitis. Conversely, the MEgreen module (*r* = 0.85, *p*=3e − 06), MEmagenta module (*r* = 0.65, *p*=0.002), MEmagenta module (*r* = 0.58, *p*=0.008), MEmidnightblue module (*r* = 0.55, *p*=0.01), and MEcyan module (*r* = 0.47, *p*=0.04) were negatively correlated with periodontitis (Figure 2c). Further analysis of genes within these four modules revealed 3538 genes negatively correlated with periodontitis (Supporting Information 6: Table S5).

### 3.4. SMR, Differential Expression Analysis, and WGCNA Collectively Identified Genes Associated with Periodontitis

To further ascertain genes related to periodontitis, we took the intersection of genes obtained from the aforementioned steps. Specifically, we intersected genes positively associated with periodontitis identified in SMR, and genes significantly upregulated in periodontitis from the differential expression analysis, and genes positively correlated with periodontitis from WGCNA, resulting in four genes (Figure 2d). Simultaneously, we intersected genes negatively associated with periodontitis identified in SMR, and genes significantly downregulated in periodontitis from the differential expression analysis, and genes negatively correlated with periodontitis from WGCNA, yielding six genes (Figure 2e). Therefore, we identified a total of 10 genes associated with periodontitis for further construction of diagnostic models using machine learning algorithms.

### 3.5. Identification of Potential Biomarkers

**Figure 3:**
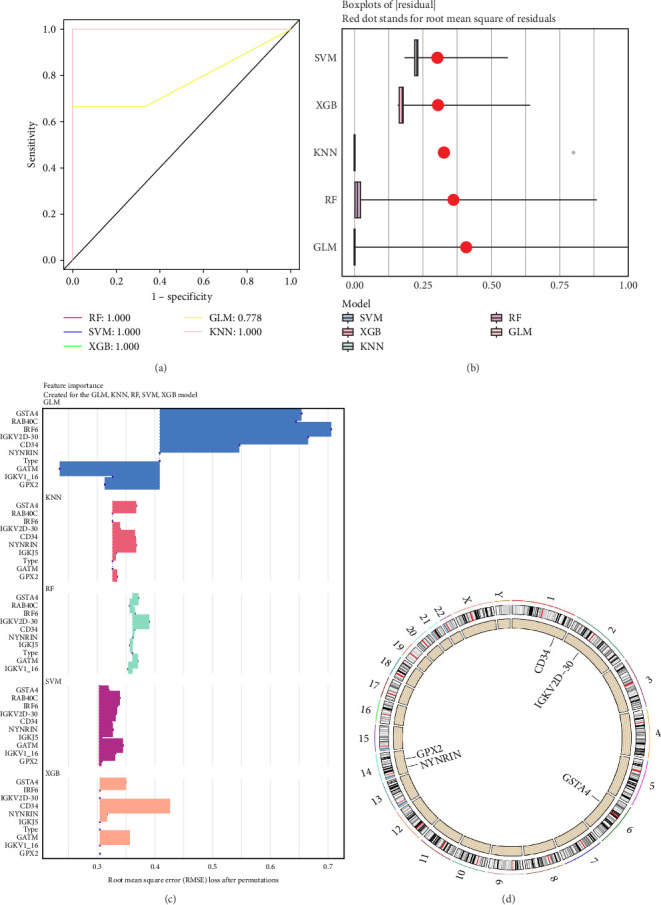
Identification of potential biomarkers for periodontitis using machine learning (ML) algorithms. (a) AUC comparison of models employing various ML classification algorithms. (b) Residual comparison across different models. (c) Top 5 most significant genes identified by different ML classification algorithms. (d) Chromosomal locations of the potential biomarkers.

We further utilized five machine learning classification methods to screen the previously identified marker genes. Among these five machine learning models, RF, SVM, XGB, and KNN all yielded excellent AUC values (1.00) in the training set (Figure 3a). We further calculated the residual values of the models in the validation set; the results showed that the KNN model had the lowest residual value (Figure 3b). Finally, we identified the five most significant genes (GPX2, IGKV2D-30, CD34, GSTA4, and NYNRIN) recognized by KNN as potential biomarkers (Figure 3c). Additionally, we determined the chromosomal locations of these biomarkers: GPX2 and NYNRIN are located on chromosome 14, IGKV2D-30 on chromosome 2, CD34 on chromosome 1, and GSTA4 on chromosome 6 (Figure 3d).

### 3.6. Identification of Potential Biomarkers

To identify robust biomarkers for periodontitis, we applied five machine learning classification algorithms—RF, SVM, XGB, KNN, and LR—to evaluate the predictive performance of the previously identified marker genes. As shown in Figure 3a, RF, SVM, XGB, and KNN all achieved perfect classification performance in the training set, with area AUC values reaching 1.00, while LR exhibited slightly lower performance. To avoid overfitting, we further compared model residuals using the validation set. The KNN model demonstrated the lowest residual error, indicating superior generalizability and stability (Figure 3b). Next, we compared the top-ranked genes identified by each algorithm and observed substantial overlap across methods. Notably, KNN consistently highlighted five genes—GPX2, IGKV2D-30, CD34, GSTA4, and NYNRIN—as the most significant contributors to model performance (Figure 3c), suggesting their potential as candidate biomarkers. We further mapped their chromosomal locations: GPX2 and NYNRIN on chromosome 14, IGKV2D-30 on chromosome 2, CD34 on chromosome 1, and GSTA4 on chromosome 6 (Figure 3d). These genes have been previously implicated in oxidative stress (GPX2 and GSTA4), immune regulation (CD34 and IGKV2D-30), and RNA processing (NYNRIN), which are all biologically relevant to the inflammatory and immune mechanisms underlying periodontitis.

### 3.7. Causal Effects of Potential Biomarkers on Periodontitis

**Figure 4:**
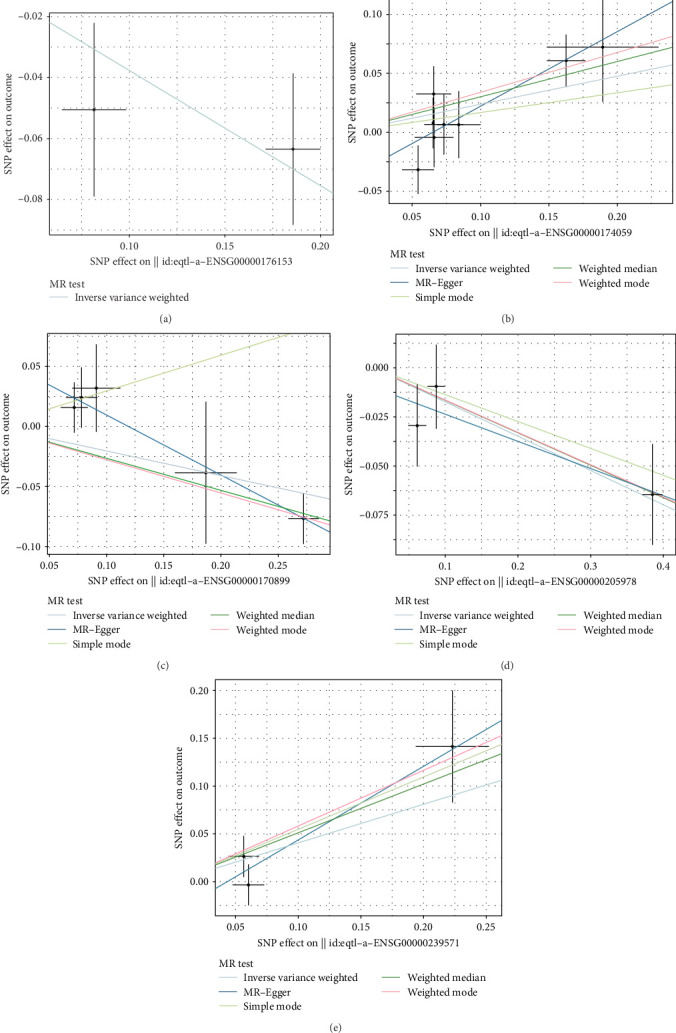
Scatter plots of causality. Scatter plots of causality of potential biomarkers GPX2 (a), CD34 (b), GSTA4 (c), NYNRIN (d), and IGKV2D-30 (e) on periodontitis using univariable Mendelian randomization.

**Figure 5:**
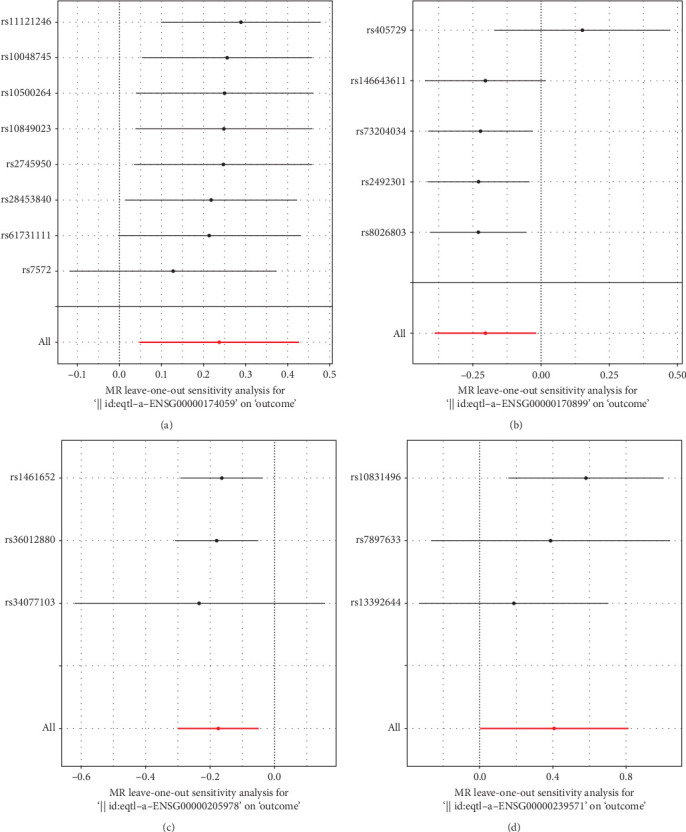
Leave one out of sensitivity tests. Calculate the MR results of the remaining IVs after removing the IVs one by one. Exposure: (a) CD34, (b) GSTA4, (c) NYNRIN, and (d) IGKV2D-30.

To further explore whether the identified biomarkers play a causal role in periodontitis, we performed TSMR analysis using eQTL data as the exposure and GWAS summary data for periodontitis as the outcome. As shown in Figure 4 and detailed in Supporting Information 7: Table S6, five genes—GPX2, CD34, GSTA4, NYNRIN, and IGKV2D-30—demonstrated significant causal associations with periodontitis. For GPX2, two instrumental SNPs were used in the MR analysis, yielding a significant inverse association (IVW: OR = –0.377, 95% CI: –0.621 to –0.133, *p*=0.002), suggesting a protective effect of increased GPX2 expression. CD34, analyzed with eight instrumental SNPs, showed a positive causal effect (OR = 0.237, 95% CI: 0.047–0.427, *p*=0.014), indicating that higher CD34 expression may contribute to elevated periodontitis risk. Similarly, GSTA4 (5 SNPs, OR = –0.205, 95% CI: –0.391 to –0.020, *p*=0.031), NYNRIN (5 SNPs, OR = –0.174, 95% CI: –0.298 to –0.050, *p*=0.006), and IGKV2D-30 (3 SNPs, OR = –0.405, 95% CI: –0.809 to –0.002, *p*=0.049) were all inversely associated with periodontitis, implying potential protective roles. To ensure robustness, we assessed heterogeneity using Cochran's *Q* test for each gene, and no significant heterogeneity was detected across IVs (all *p*  > 0.05; Supporting Information 8: Table S7), supporting the validity of the IVW estimates. In addition, no evidence of horizontal pleiotropy was detected (Supporting Information 11: Table S8). Moreover, the *F*-statistics for all selected SNPs exceeded the threshold of 10, indicating sufficient instrument strength and reducing the likelihood of weak instrument bias. Leave-one-out sensitivity analyses (Figure 5) did not identify any single SNP disproportionately influencing the overall estimates, further reinforcing the stability of the causal inferences. Figure 4 presents scatter plots showing SNP-specific effect sizes for gene expression (exposure) and periodontitis (outcome), with fitted lines for IVW and MR–Egger estimates. These plots reveal a consistent direction of effect across SNPs for each gene, visually supporting the statistical findings. Notably, the protective genes (GPX2, GSTA4, NYNRIN, and IGKV2D-30) all showed leftward trends (negative slopes), while CD34 displayed a rightward trend (positive slope), clearly distinguishing their biological implications.

Together, these results strengthen the biological plausibility of the five machine learning-prioritized genes and suggest that they may not only serve as diagnostic biomarkers but also participate causally in the development or progression of periodontitis. These findings provide an essential foundation for future functional validation and therapeutic targeting.

### 3.8. Construction and Validation of the Nomogram

**Figure 6:**
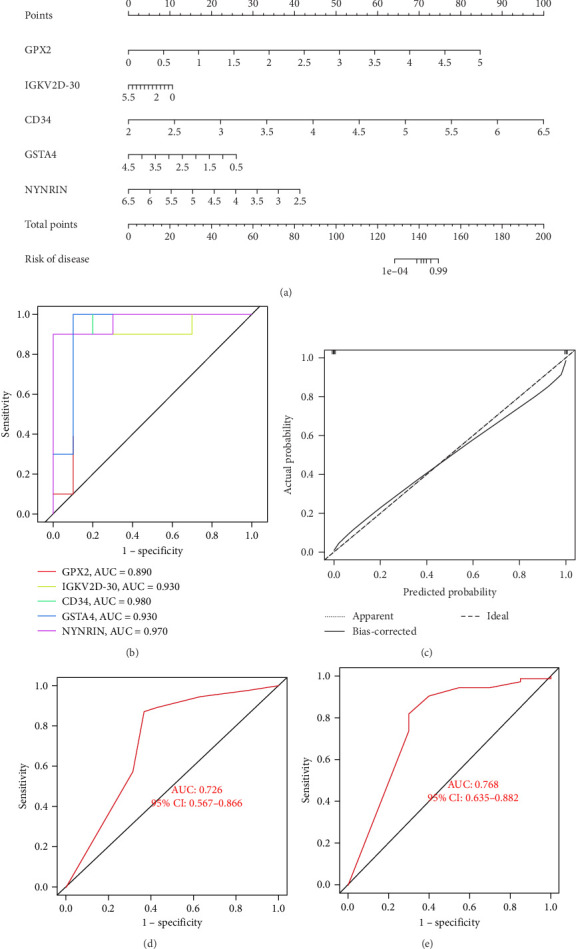
Nomogram construction and diagnostic value assessment. (a) The constructed nomogram for periodontitis diagnosis. (b) ROC curve for potential biomarkers in the training dataset. (c) Calibration curve demonstrating the model's performance. (d) ROC curve for the diagnostic model in the GSE10334 dataset. (e) ROC curve for the diagnostic model in the GSE16134 dataset.

We constructed a diagnostic model using logistic regression based on the expression of potential biomarkers in the training set. The model has been visualized as a diagnostic nomogram in Figure 6a. Additionally, ROC analysis was conducted to compare the predictive accuracy of the model. We observed that our model outperformed other single biomarker models, with the highest AUC value of gene reaching 0.980 (Figure 6b). Furthermore, calibration curves demonstrated excellent predictive capability of the model (Figure 6c). Finally, we validated these results using the validation set (Figure 6d).

### 3.9. Assessment of Potential Biomarkers Expression Levels

**Figure 7:**
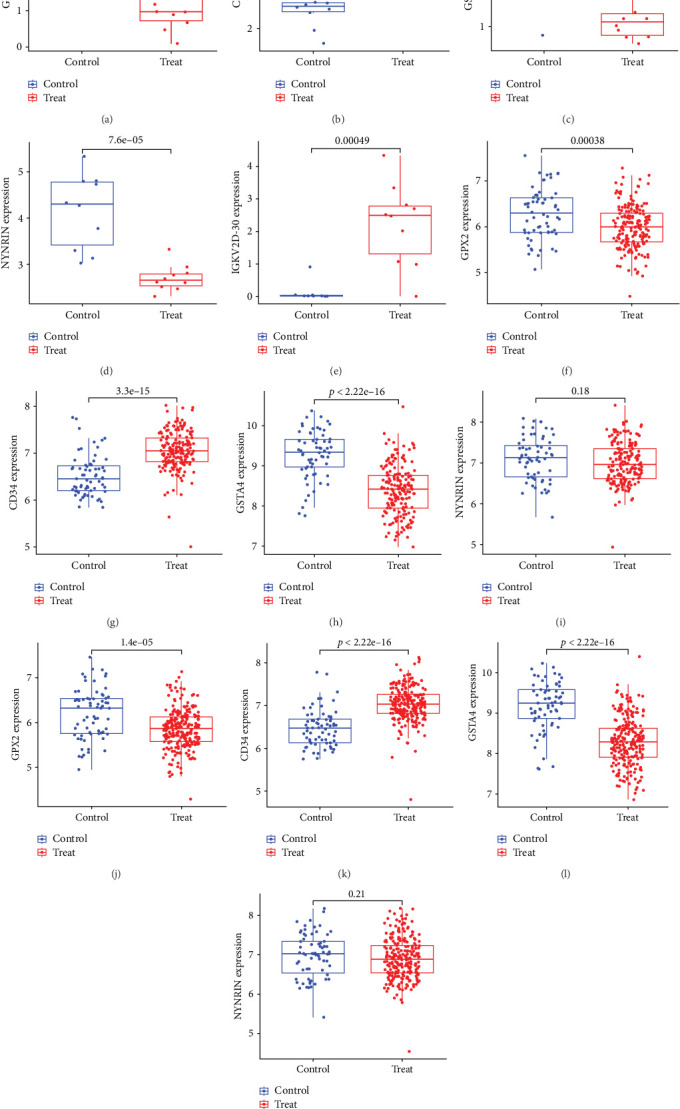
Evaluation of the expression levels of the potential biomarkers of periodontitis in the training and validation sets. (a–e) The expression levels of GPX2 (a), CD34 (b), GSTA4 (c), NYNRIN (d), and IGKV2D-30 (e) in training set. (f–i) The expression levels of GPX2 (f), CD34 (g), GSTA4 (h), and NYNRIN (i) in GSE10334 dataset. (j–m) The expression levels of GPX2 (j), CD34 (k), GSTA4 (l), and NYNRIN (m) in GSE16134 dataset.

We further analyzed the expression levels of five biomarkers in patients with periodontitis. The corresponding results revealed that, compared to control samples, IGKV2D-30 and CD34 were downregulated in patients with periodontitis, while GPX2, GSTA4, and NYNRIN were upregulated in patients with periodontitis (Figure 7). These results were similar in the validation set; however, the expression of NYNRIN showed no statistical difference between the NOA group and the control group, and IGKV2D-30 was not detected in the validation set (Figure 7).

### 3.10. Biological Functions of Potential Biomarkers

Subsequently, we analyzed the signaling pathways associated with the five potential biomarkers. GSVA results indicated that the high expression of IGKV2D-30 and CD34 was mainly enriched in signaling pathways, such as flavonoid metabolic process, xenobiotic glucuronidation, drug metabolism other enzymes, steroid hormone biosynthesis, retinol metabolism, porphyrin and chlorophyll metabolism, among others. Conversely, the low expression of NYNRIN was predominantly enriched in signaling pathways, including starch and sucrose metabolism, porphyrin and chlorophyll metabolism, steroid hormone biosynthesis, drug metabolism other enzymes, ascorbate and aldarate metabolism (Supporting Information 9: Figure S2).

Additionally, we conducted GSEA analysis on these genes. The results revealed that the high expression of IGKV2D-30 and CD34 was enriched in pathways, such as chemokine signaling pathway, focal adhesion, systemic lupus erythematosus, et cetera. Conversely, the low expression of NYNRIN was enriched in pathways, including systemic lupus erythematosus, N-glycan biosynthesis, among others. The low expression of GPX2 was enriched in pathways, such as metabolism of xenobiotics by cytochrome P450, pentose and glucuronate interconversions, and retinol metabolism. Similarly, the low expression of GSTA4 was enriched in pathways, like chemokine signaling pathway, cytokine–cytokine receptor interaction, hematopoietic cell lineage, oxidative phosphorylation, systemic lupus erythematosus, among others. (Supporting Information 10: Figure S3).

### 3.11. Evaluation of Immune Cell Infiltration

**Figure 8:**
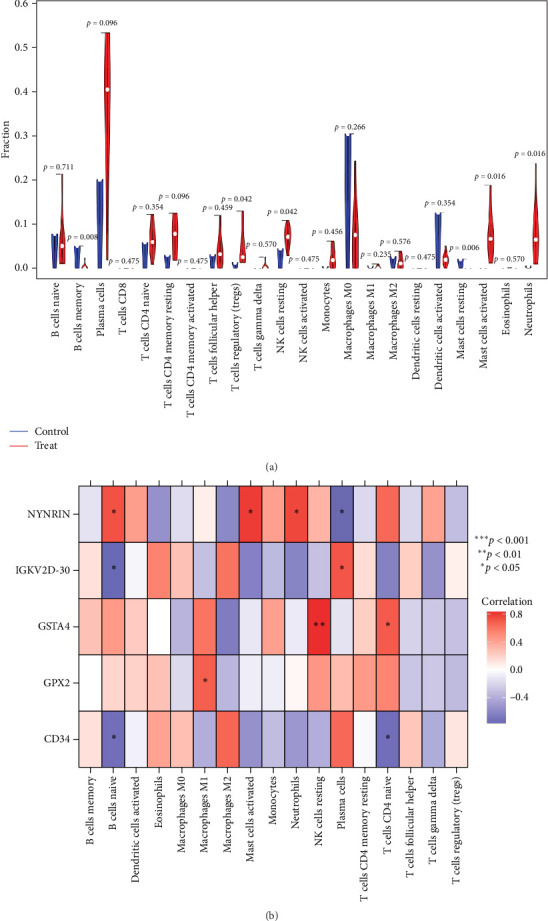
Immune microenvironment of gingival tissue in periodontitis patients and healthy controls. (a) Heat map of correlation between potential markers and immune cells. (b) Differences of immune cells composition between periodontitis patients and healthy controls in training set.

The CIBERSORT algorithm was employed to evaluate the levels of immune cell infiltration in both periodontitis and normal healthy groups. CIBERSORT analysis revealed that T cells regulatory (Tregs), activated mast cells, and neutrophils were significantly higher in periodontitis patients compared to normal healthy individuals (Figure 8a). However, the levels of memory B cells and resting mast cells were notably lower in periodontitis patients compared to normal healthy individuals (Figure 8a). Subsequently, we also computed the correlation between potential biomarkers and immune cell content. NYNRIN was positively correlated with naive B cells, activated mast cells, and neutrophils, while negatively correlated with plasma cells. IGKV2D-30 was positively correlated with plasma cells and negatively correlated with naive B cells. GPX2 was positively correlated with macrophages M1. CD34 was negatively correlated with naive B cells and naive CD4 T cells (*p*  < 0.05; Figure 8b).

## 4. Discussion

In this study, we conducted SMR analysis on the blood eQTL dataset, considering it as the exposure factor, with periodontitis as the outcome. We identified 294 genes that may have a causal relationship with severe periodontitis. GO enrichment analysis of these genes indicated that the development of periodontitis might involve biological processes, such as lymph vessel morphogenesis, regulation of metaphase plate congression, vesicle transport along microtubules, and regulation of interleukin-2 production. These processes are likely related to immune responses, cell proliferation, and inflammatory responses, which are central to the pathogenesis of periodontitis. Additionally, we assessed the gene expression profiles of periodontitis samples from the GEO database and identified 2097 DEGs between control and periodontitis samples. GO enrichment analysis of these DEGs suggested alterations in immune system activation and regulation, as well as potential impacts on nervous system and skin development in periodontitis patients compared to healthy individuals. These changes may play crucial roles in the pathogenesis of periodontitis. WGCNA has been successfully applied in previous studies to evaluate the associations between gene modules and clinical traits, enabling the identification of key genes associated with specific traits. Through WGCNA, we identified 5817 genes associated with periodontitis. Based on this analysis, we obtained 10 provisional overlapping genes. Subsequently, we used five different machine learning algorithms to identify potential biomarkers. Machine learning, an emerging interdisciplinary field, plays a significant role in various aspects of medicine. Ultimately, using the KNN algorithm, we identified GPX2, IGKV2D-30, CD34, GSTA4, and NYNRIN as potential biomarkers, which showed the lowest residual values. Additionally, TSMR confirmed a significant causal relationship between these genes and periodontitis.

In patients with periodontitis, the expression of IGKV2D-30 and CD34 is downregulated. IGKV2D-30 is a component of the immunoglobulin kappa light chain [46]. The immunoglobulin kappa light chain plays a crucial role in the immune system by recognizing and eliminating pathogens, thereby maintaining immune function [47]. In periodontitis, the downregulation of IGKV2D-30 may impair the immune response in gingival tissue, resulting in increased immune deficiency, bacterial infection, and immune tolerance. Therefore, the abnormal expression of IGKV2D-30 may negatively impact the immune health of patients with periodontitis. Simultaneously, some studies have found a reduced number of CD34-positive stem cells in the gingival tissue of periodontitis patients [48]. CD34-positive stem cells are vital for tissue repair and regeneration. Their reduction may hinder the repair of gingival tissue in periodontitis patients, preventing the restoration of normal structure and function, and exacerbating the progression of periodontitis. On the other hand, we found that peroxidase (GPX2) and glutathione S-transferase (GSTA4) are upregulated in the gingival tissue of periodontitis patients. This upregulation is associated with oxidative stress response and bacterial infection in periodontitis tissue. GPX2 and GSTA4 are important antioxidant enzymes, and their upregulation may represent an adaptive response to oxidative damage and detoxification of harmful substances [49–51]. During periodontitis, cells are affected by oxidative stress and bacterial infection, leading to the accumulation of toxic metabolic products. GPX2 and GSTA4 protect cells from oxidative damage by catalyzing the reduction of harmful substances, such as hydrogen peroxide. Therefore, their upregulation may help maintain intracellular oxidative balance and mitigate oxidative stress-induced damage in periodontitis tissue. Lastly, we found an upregulation trend of NYNRIN in the gingival tissue of periodontitis patients. Although its specific role is not yet fully understood, some studies suggest that NYNRIN may be involved in neuronal development and function [52, 53]. In periodontitis, neuronal activation and damage are potential occurrences. Chronic inflammation and bacterial infection may directly stimulate peripheral neurons, leading to neuronal activation and functional changes, including oxidative damage and apoptosis. Therefore, the upregulation of NYNRIN might be part of a neuroprotective mechanism. In summary, although the specific function of NYNRIN is not yet completely clear, its upregulation may be related to neuronal development and function.

Furthermore, immune infiltration analysis revealed significant immune alterations in periodontitis tissue. Compared to healthy controls, Tregs, activated mast cells, and neutrophils were significantly elevated, whereas memory B cells and resting mast cells were decreased. The increased Tregs may indicate the body's attempt to mitigate excessive immune activation and control inflammation [54]. Elevated activated mast cells and neutrophils suggest an active inflammatory state, where these cells release mediators and enzymes that promote tissue damage [55, 56]. In contrast, memory B cells are critical for long-term antigen recognition and immune memory [57], and their reduction may impair the host's ability to resist recurrent microbial invasion. Resting mast cells, known for regulating inflammation and tissue repair, were also decreased, possibly contributing to immune dysregulation and impaired homeostasis [58]. These findings underscore the pivotal role of immune cell function and immune homeostasis in the pathogenesis of periodontitis. They are in line with recent studies that have demonstrated causal links between circulating immune proteins, immune cells, and periodontal traits [59, 60], highlighting systemic immune regulation as a potential therapeutic target. Collectively, our integrative approach—combining genetic causal inference, expression profiling, immune cell landscape analysis, and machine learning-based biomarker discovery—provides new insights into the immunopathogenesis of periodontitis and offers a foundation for the development of novel diagnostic and therapeutic strategies.

Our study has several notable strengths. First, we employed multi-SNP SMR analysis for sensitivity assessment, providing more robust evidence and minimizing the false-positive rate. Second, we integrated multiomics datasets, including GWAS, eQTL, and machine learning-based transcriptomic analysis, which improved the comprehensiveness and reliability of biomarker identification. Third, the use of multiple machine learning algorithms allowed for stable feature selection and enhanced interpretability. However, several limitations should be acknowledged. First, although GWAS offers a powerful approach for discovering genetic associations, it may not account for all potential confounding factors. Nonetheless, the use of eQTL data and MR design helps mitigate confounding and reverse causation. Second, despite conducting sensitivity analyses, we cannot completely rule out horizontal pleiotropy, which may still bias causal estimates. Third, and most importantly, although we systematically identified and prioritized candidate genes through bioinformatics and computational approaches, we acknowledge the lack of experimental validation as a major limitation. The biological functions and mechanistic roles of the predicted genes—such as GPX2, CD34, and GSTA4—in the pathogenesis of periodontitis remain to be empirically confirmed. Future studies should focus on validating these biomarkers through in vitro experiments, such as overexpression or knockdown of candidate genes in periodontal ligament cells, gingival epithelial cells, or macrophage models to assess their effects on inflammatory cytokine expression, oxidative stress responses, and tissue remodeling. Such functional validation will not only confirm the causal relevance of these genes but also provide insights into potential therapeutic targets and mechanisms. Therefore, our findings offer a valuable framework and hypothesis-generating resource for further experimental investigations.

## 5. Conclusion

In this study, we identified 294 genes with potential causal relationships to severe periodontitis through eQTL-based analysis. Functional enrichment revealed that these genes are involved in immune response, inflammation, and cell proliferation. Moreover, we identified GPX2, IGKV2D-30, CD34, GSTA4, and NYNRIN as candidate biomarkers with both differential expression and genetic evidence of causality. These findings deepen our understanding of the immunogenetic mechanisms underlying periodontitis and highlight potential targets for diagnosis and therapy.

## Data Availability

FinnGen project (https://www.finngen.fi/en), eQTL-Gen database (https://eqtlgen.org/), and GEO database (https://www.ncbi.nlm.nih.gov/).

## Supporting Information

Supporting Information 1 Figure S1. Study flowchart. The flowchart summarizes the overall study design and analysis pipeline.

Supporting Information 2 Table S1. Summary statistics of the transcriptome-wide SMR analysis with periodontitis.

Supporting Information 3 Table S2. Differentially expressed genes between periodontitis and normal gingival tissues.

Supporting Information 4 Table S3. GO enrichment analysis of significantly highly expressed genes in periodontitis gingival tissues compared to normal gingival tissues.

Supporting Information 5 Table S4. GO enrichment analysis of significantly lowly expressed genes in periodontitis gingival tissues compared to normal gingival tissues.

Supporting Information 6 Table S5. Genes associated with periodontitis identified by WGCNA.

Supporting Information 7 Table S6. Causal effect of potential biomarkers on periodontitis using univariable Mendelian randomization.

Supporting Information 8 Table S7. Heterogeneity test of potential biomarkers and periodontitis.

Supporting Information 9 Figure S2. GSVA analysis of high and low expression of Apotential biomarker. (A–E) GSVA by “c5.go.symbols.gmt” of GPX2 (A), CD34 (B), GSTA4 (C), NYNRIN (D), and IGKV2D-30 (E). (F–J) GSVA by “c2.cp.kegg.symbols.gmt”of GPX2 (F), CD34 (G), GSTA4 (H), NYNRIN (I), and IGKV2D-30 (J).

Supporting Information 10 Figure S3. GSEA analysis of high and low expression of Apotential biomarker. (A–E) GSEA by “c5.go.symbols.gmt” of GPX2 (A), CD34 (B), GSTA4 (C), NYNRIN (D), and IGKV2D-30 (E). (F–J) GSEA by “c2.cp.kegg.symbols.gmt”of GPX2 (F), CD34 (G), GSTA4 (H), NYNRIN (I), and IGKV2D-30 (J).

Supporting Information 11 Table S8. Horizontal pleiotropy results of potential biomarkers and periodontitis.
