# Supplementary figures and images for: Causal Gene Identification and Biomarker Prioritization in Periodontitis via Integrative Multiomics and Mendelian Randomization
Source: Mediators Inflamm. 2025 Nov 24;2025:6044837. doi: 10.1155/mi/6044837 (PMC12668852; doi:10.1155/mi/6044837)

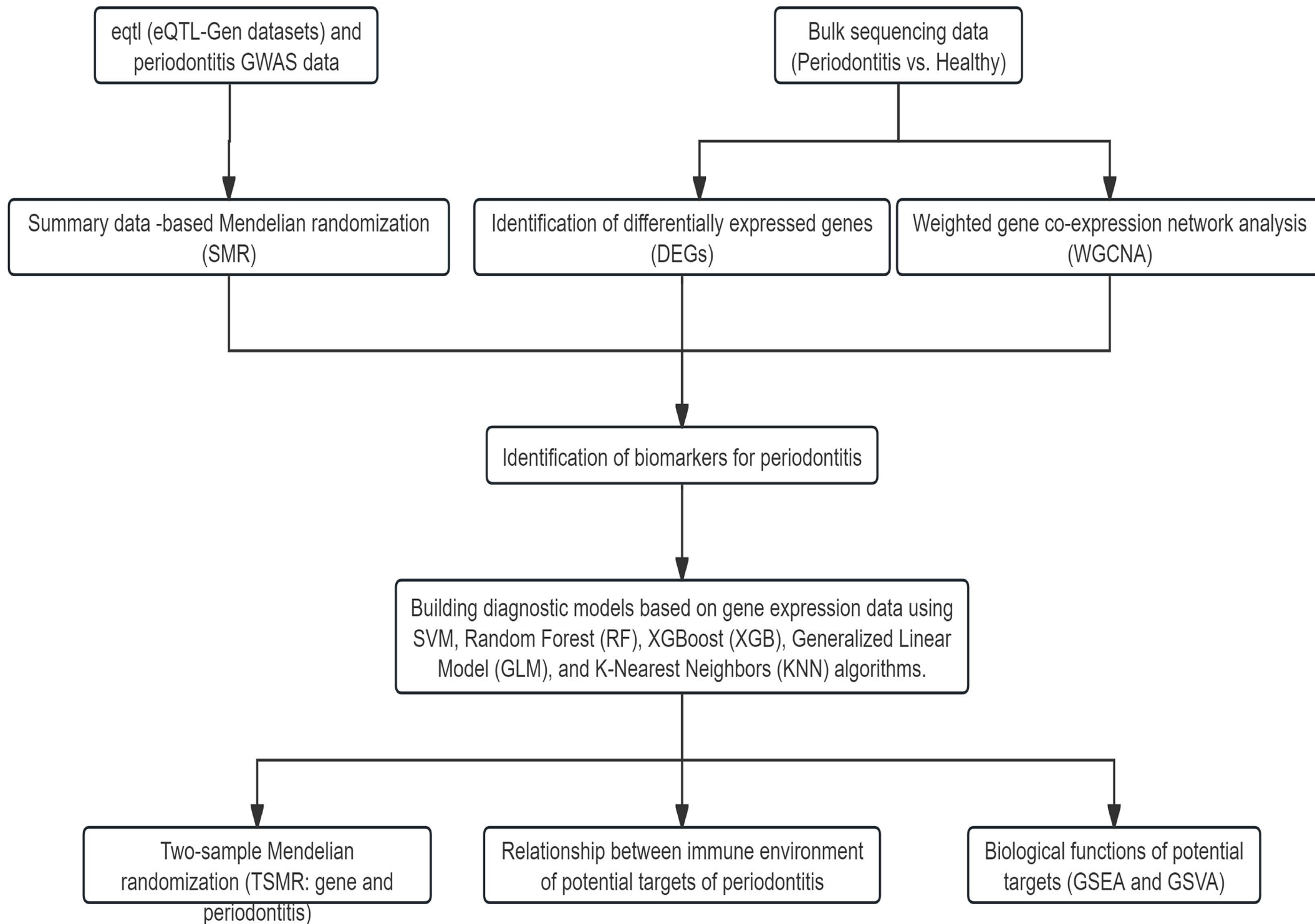

Supplement: Supporting Information 1 — Figure S1. Study flowchart. The flowchart summarizes the overall study design and analysis pipeline. [file 6044837.f1.pdf]

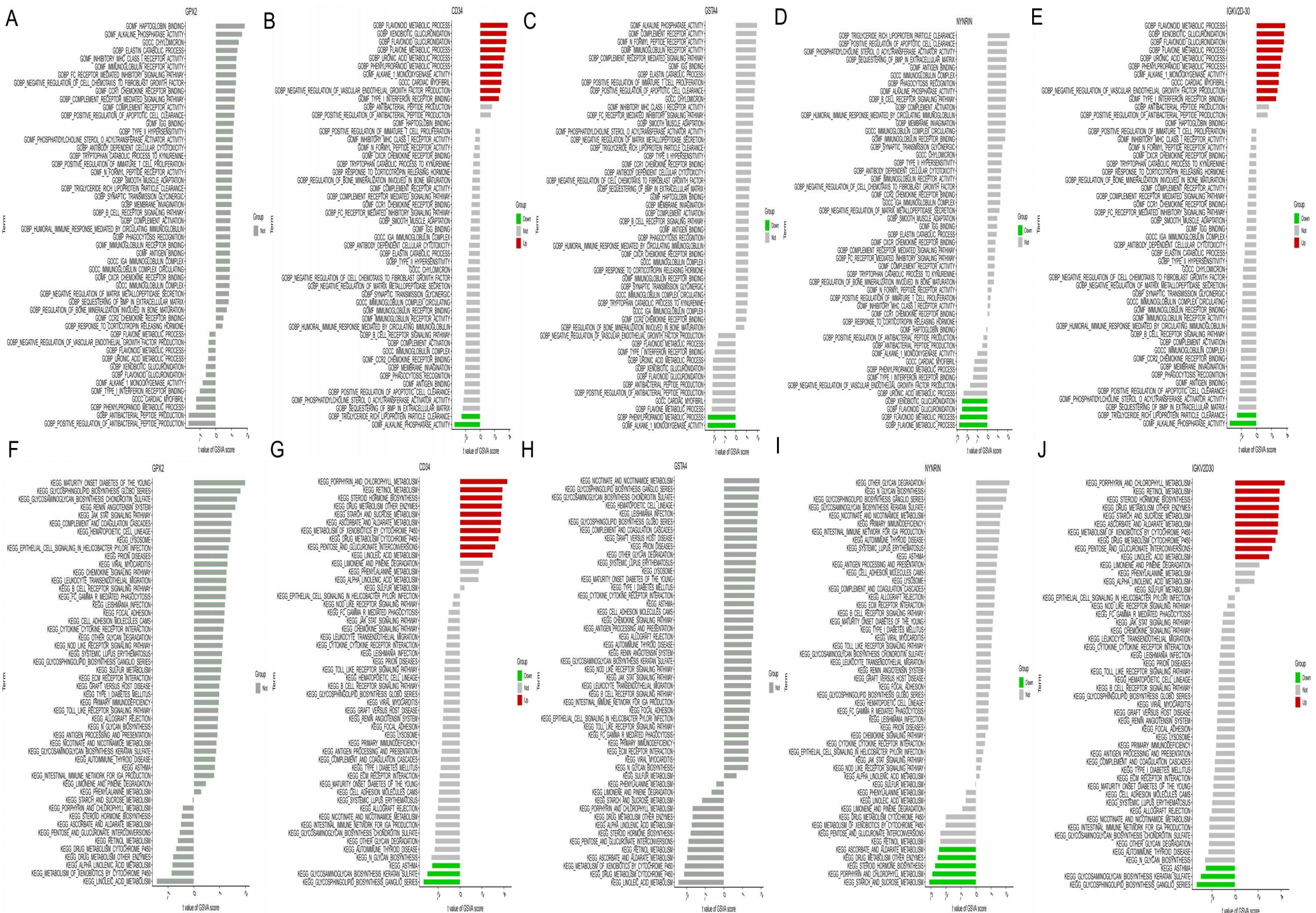

Supplement: Supporting Information 9 — Figure S2. GSVA analysis of high and low expression of Apotential biomarker. (A–E) GSVA by “c5.go.symbols.gmt” of GPX2 (A), CD34 (B), GSTA4 (C), NYNRIN (D), and IGKV2D-30 (E). (F–J) GSVA by “c2.cp.kegg.symbols.gmt”of GPX2 (F), CD34 (G), GSTA4 (H), NYNRIN (I), and IGKV2D-30 (J). [file 6044837.f9.pdf]

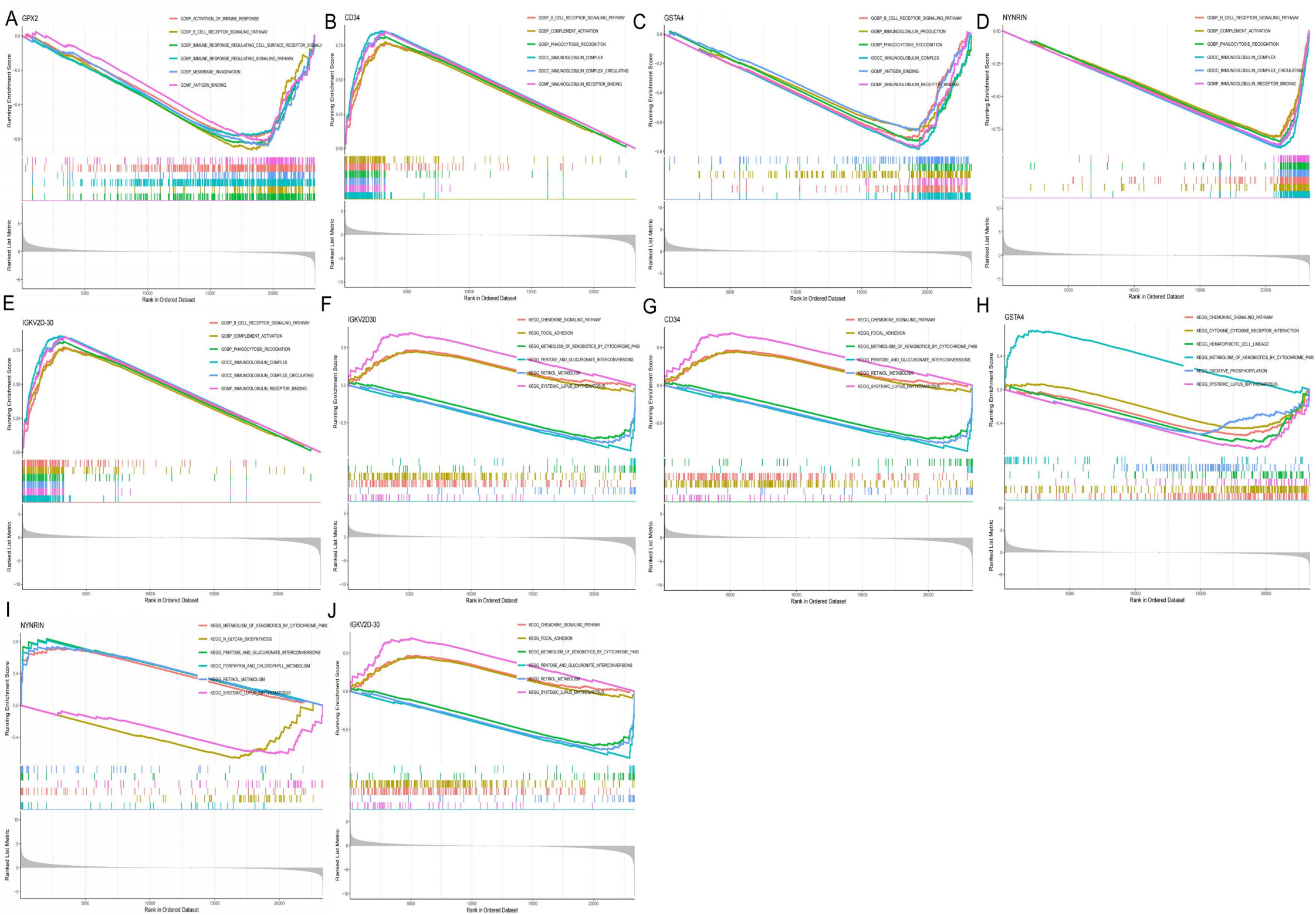

Supplement: Supporting Information 10 — Figure S3. GSEA analysis of high and low expression of Apotential biomarker. (A–E) GSEA by “c5.go.symbols.gmt” of GPX2 (A), CD34 (B), GSTA4 (C), NYNRIN (D), and IGKV2D-30 (E). (F–J) GSEA by “c2.cp.kegg.symbols.gmt”of GPX2 (F), CD34 (G), GSTA4 (H), NYNRIN (I), and IGKV2D-30 (J). [file 6044837.f10.pdf]
